# A Novel Binary T-Vector with the *GFP* Reporter Gene for Promoter Characterization

**DOI:** 10.1371/journal.pone.0107328

**Published:** 2014-09-08

**Authors:** Shu-Ye Jiang, Jeevanandam Vanitha, Yanan Bai, Srinivasan Ramachandran

**Affiliations:** Temasek Life Sciences Laboratory, the National University of Singapore, Singapore, Singapore; National Key Laboratory of Crop Genetic Improvement, China

## Abstract

Several strategies have been developed to clone PCR fragments into desired vectors. However, most of commercially available T-vectors are not binary vectors and cannot be directly used for *Agrobacterium*-mediated plant genetic transformation. In this study, a novel binary T-vector was constructed by integrating two *Ahd*I restriction sites into the backbone vector pCAMBIA 1300. The T-vector also contains a *GFP* reporter gene and thus, can be used to analyze promoter activity by monitoring the reporter gene. On the other hand, identification and characterization of various promoters not only benefit the functional annotation of their genes but also provide alternative candidates to be used to drive interesting genes for plant genetic improvement by transgenesis. More than 1,000 putative pollen-specific rice genes have been identified in a genome-wide level. Among them, 67 highly expressed genes were further characterized. One of the pollen-specific genes *LOC_Os10g35930* was further surveyed in its expression patterns with more details by quantitative real-time reverse-transcription PCR (qRT-PCR) analysis. Finally, its promoter activity was further investigated by analyzing transgenic rice plants carrying the promoter::*GFP* cassette, which was constructed from the newly developed T-vector. The reporter *GFP* gene expression in these transgenic plants showed that the promoter was active only in mature but not in germinated pollens.

## Introduction

Several strategies have been explored to clone DNA fragments from polymerase chain reaction (PCR) into desired vectors. One of the strategies is to incorporate restriction enzyme sites into oligonucleotide primers to create sticky ends by digesting the PCR products. However, the digestion efficiency is low for many restriction endonucleases when their recognition sequences are located within a few base pairs of the end of the PCR products [1]. The second strategy is to clone PCR products as blunt-ended fragments. The strategy requires an enzymatic processing to remove a 3′ overhang adenosine which was generated by PCR Taq polymerase due to its template-independent terminal transferase activity [2]. The third strategy is to directly clone PCR products into a T-vector with 3′-T overhang. The strategy is applicable to clone PCR products with 3′-T overhang generated by some of thermostable DNA polymerases.

A T-vector can be constructed by two ways. One way is to artificially add T-overhang to blunt-ended plasmid DNA by terminal deoxynucleotidyl-transferase (TdT) or Taq polymerase in the presence of dideoxythymidine triphosphate (ddTTP) and dTTP, respectively [3]–[5]. By this way, the prerequisite is that one should first construct a plasmid DNA with blunt-ended restriction enzyme sites. Another way is to directly generate a 3′ overhang linear vector by certain restriction enzymes. Several restriction enzymes could be used for that purpose and the examples include *Bci*VI, *Bfi*I, *Hph*I, *Mnl*I, *Taa*I, *Xcm*I and *Ahd*I (*Eam*1105I) [6]–[9]. The widely employed enzyme is *Xcm*I. The enzyme has a flexible recognition sites “CCANNNNN/NNNNTGG” (N = A/C/T/G). Thus, a plasmid DNA with the sequence “CCANNNNTNNNNTGG” can be recognized by *Xcm*I and be digested to generate a 3′ overhang linear vector. Currently, considerable reports have been published to introduce the application of this enzyme in the generation of 3′ overhang vectors for cloning PCR products [7], [10]–[12]. Besides *Xcm*I, little is known about the application of the other enzymes on generating the 3′ overhang vector. In addition, most of commercially available T-vectors are not binary vectors and cannot be directly used for *Agrobacterium*-mediated genetic transformation. In this study, we have constructed a novel binary T-vector by inserting a 1,317-bp DNA sequence with two *Ahd*I restriction sites. The T-vector contains a *GFP* reporter gene and thus, can be used to analyze promoter functions by the *GFP* gene.

Identification and characterization of various promoters not only benefit the functional annotation of their genes but also provide alternative candidates to be used to drive interesting genes for genetic improvement of targeted organisms by transgenesis. Among different types of promoters, we are interested in pollen-specific promoters. These promoters can be used to drive expression of some genes to disturb pollen development by genetic transformation and as a result, to develop male sterile lines. Genetically stable male sterile lines are the prerequisite to commercially utilize crop heterosis to improve crop production. Thus, pollen-specific promoters are potentially useful in crop genetic improvement by developing hybrid crop cultivars [13]–[15].

Some of pollen-specific genes and their promoters were isolated and characterized more than twenty years ago from plants [16], [17]. Since then, more numbers of pollen-specific promoters have been characterized and these include petunia *PA2* [18], tomato *LAT52* and *LAT59* [19], [20], rapeseed *Bp10* [21], maize *Zm13* [22], [23], tobacco *NTP303* [24], Lily *cgH3* [25] and *Arabidopsis* promoters *TUA1* [26], *AtPTEN1* [27], *AtSTP6* [28], *AtSTP9* [29] and *AtVEX1* [30]. Among them, the tomato *LAT52* promoter has been widely used to drive pollen-specific expression. In rice, promoters from numbers of genes showed activities in mature pollens and/or pollen tubes and examples include *Osnop*, *OsSCP1*, *OsSCP2*, *OsSCP3*, *OSIPA*, *OSIPK*, and *OSIPP3* [31]–[35]. Recently, Oo *et al* (2014) reported 6 genes whose promoter activities were detected in the late stage of pollen formation in the transgenic *Arabidopsis* carrying the promoter::*GUS*/*GFP* constructs [15].

Pollen development undergoes a complicated biological process from microspores to mature pollen grains. It has been regarded as an ideal model to study various biological processes such as sexual reproduction, cell fate determination, signal transduction, membrane transport, and polar growth. Pollen-specific promoters may be utilized as tools in annotating biological functions of these genes involved in pollen development and regulation. Thus, it is necessary to identify and characterize various pollen-specific promoters whose activities could be detected at only certain stage of pollen development. There are lots of publicly available rice expression databases such as Gene Expression Omnibus (GEO) datasets [36], ArrayExpress [37] and PLEXdb [38]. However, limited data are available for focusing on pollen-related expression. Besides the Massively Parallel Signature Sequencing (MPSS) rice database [39], two of microarray datasets are available where pollens and other tissues were taken for expression analysis. One of the datasets contained the expression data from 8 different samples including leaves, callus cells, roots, uninucleate microspores, bicellular pollens, tricellular pollens, mature pollen grains and germinated pollen grains [40]. Another dataset contained only 3 samples including seedlings, pollen at anthesis and sperm at anthesis [41]. All these data provide the basis for genome-wide identification of pollen-specific genes. In this study, a novel binary T-vector has been developed to facilitate the rapid clone of targeted promoters into the upstream of reporter *GFP* gene. Considerable numbers of pollen-specific rice genes were also verified in their expression. Additionally, one pollen-specific gene and its promoter were molecularly characterized by using our newly developed T-vector. The data showed that a promoter::*GFP* construct could be prepared rapidly by using the novel T-vector and the developed construct could be directly used for *Agrobacterium*-mediated genetic transformation to generate transgenic plants for detecting the promoter activities through the *GFP* reporter gene. Our data also showed that the 1,492 bp promoter from the rice gene *LOC_Os10g35930* was active only at the mature stage of pollens and no GFP activity was observed after pollen germination.

## Results

### Construction of the novel binary T-vector

**Figure 1:**
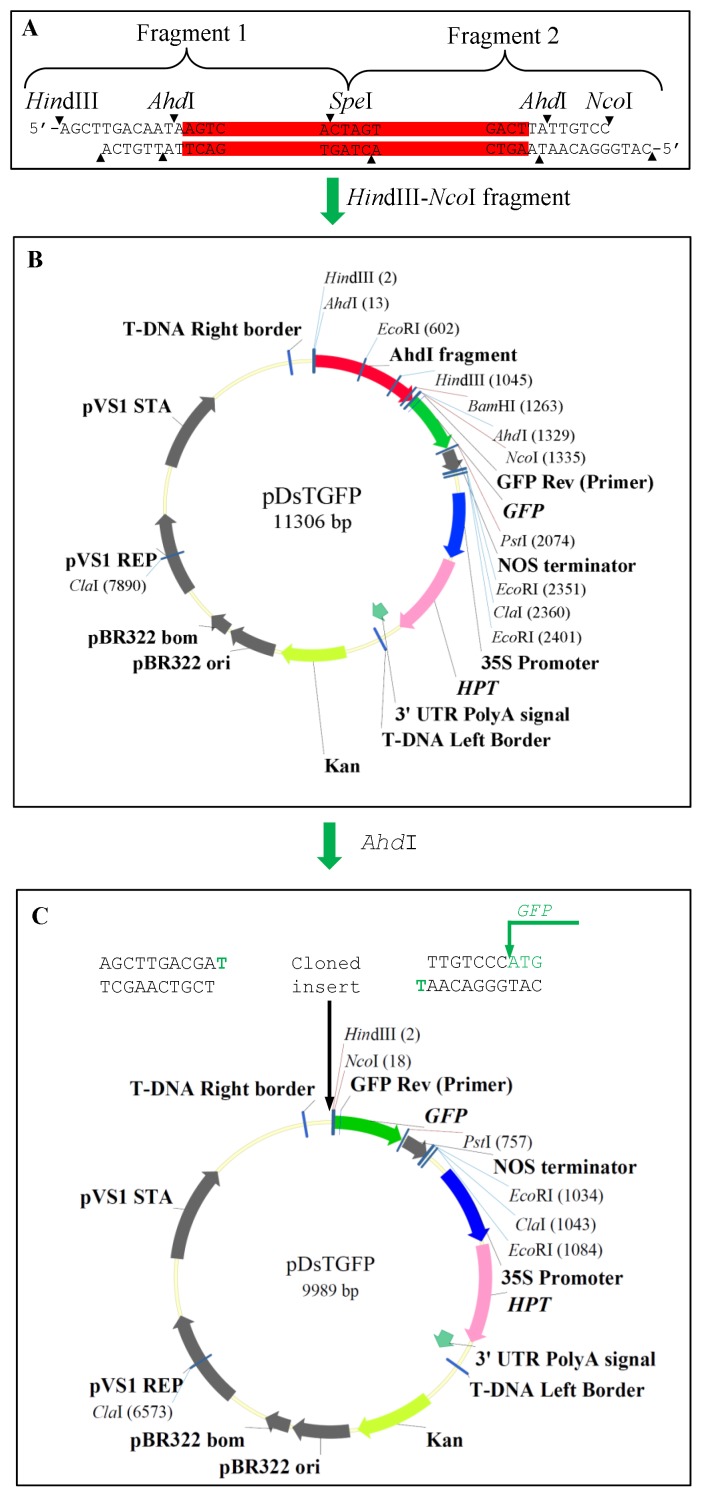
Procedure for construction of the novel T-vector. (A) Two bridge fragments were amplified from the rice genome and were then cloned into pGEM-T Easy Vector (Promega). The two fragments, ligated together by *Spe*I, contain *Hin*dIII/*Ahd*I in 5′-region and *Ahd*I/*Nco*I sites in 3′ region. The resulted *Hin*dIII-*Nco*I fragment (*Ahd*I fragment) was subcloned into the *GFP*-contaiing pCAMBIA 1300. (B) *Ahd*I fragment and *GFP* containing pCAMBIA 1300. (C) The novel T-overhang vector was produced by digesting the vector in (B) with *Ahd*I. The partial sequences with T-overhang (green letters) as cloned insert were shown above the digested vector. The start codon “ATG” of the reporter *GFP* gene was also highlighted with green color.

The restriction enzyme *Ahd*I has the recognition site GACNNN/NNGTC (N = A/C/T/G). Thus, a DNA fragment with the sequence GACAATAAGTC can be digested by the enzyme *Ahd*I to generate a 3′ T-overhang end. To introduce this sequence into the binary vector pCAMBIA 1300 (http://www.cambia.org), two bridge fragments were amplified by PCR with the rice genomic DNA as a template using two primer sets as listed in Table S1 (See Methods in details). The Fragment 1 contains two recognition sites including *Hin*dIII and *Ahd*I and the Fragment 2 has two recognition sites including *Ahd*I and *Nco*I. These two fragments were then ligated together by the enzyme *Spe*I (Figure 1A). The ligated fragment was further digested by both *Hin*dIII and *Nco*I to form a *Hin*dIII-*Nco*I fragment. The fragment was then ligated with a *GFP*-containing pCAMBIA 1300 binary vector to develop the vector named as pDsTGFP (Figure 1B). The vector has two *Ahd*I sites at the 13^th^ and 1329^th^ position, respectively (Figure 1B). Thus, the 3′ T-overhang ends can be generated by digesting the pDsTGFP vector with the enzyme *Ahd*I (Figure 1C).

### A PCR-based method for developing a promoter::*GFP* construct

**Figure 2:**
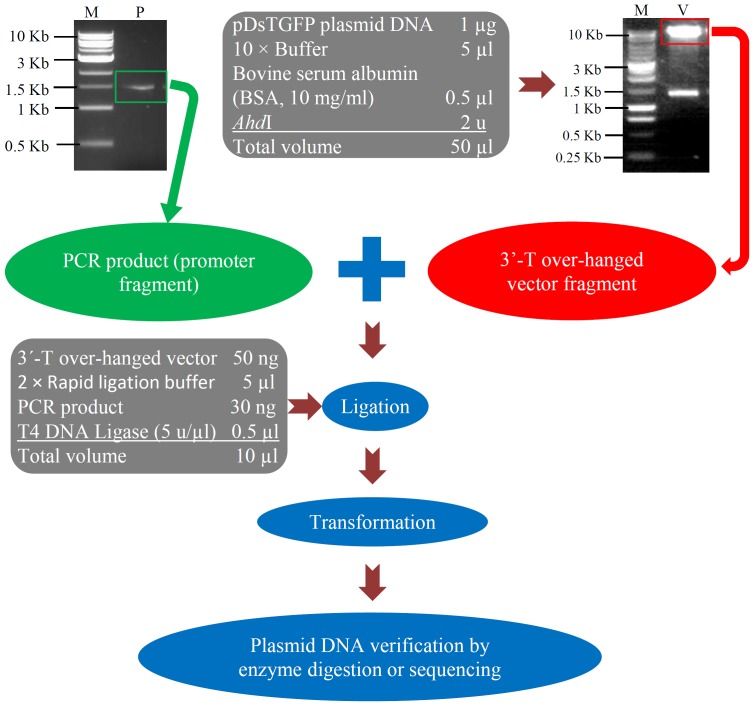
Direct PCR cloning to generate a promoter::*GFP* cassette. The figure shows the steps how to generate a promoter::*GFP* cassette for plant transgenesis. The pDsTGFP was digested by *Adh*I to produce T-overhang vector. The vector was then ligated with a PCR fragment purified from agarose gel. The ligation product was used for *Ecoli* transformation to generate the desired plasmid DNA for *Agrobacterium*-mediated transformation.

This novel binary T-vector is specially designed to clone a promoter fragment from PCR amplification using Taq DNA polymerase. For rice and many other plants, their genomes have been sequenced and annotated in a genome-wide level. A promoter sequence can be predicted by further analyzing 2 Kb upstream of a transcription start codon site of a gene. Based on the analysis, the predicted promoter sequence with 2 Kb or less than 2 Kb in length can be selected for designing promoter-specific primer sets, which will be used for PCR-amplification of the candidate promoter. An example was shown in Figure 2 (indicated by the green arrow), where an around 1.5 Kb promoter fragment was amplified. In the meantime, the plasmid DNA pDsTGFP was digested by *Ahd*I using the method as shown in Figure 2. After digestion, the products were separated by electrophoresis. The fragment with around 10 Kb (indicated by the red arrow in Figure 2) was purified from the gel, which was serviced as the 3′-overhang T-vector fragment. Both the PCR product and the 3′-overhang fragment were then ligated in the presence of T4 DNA ligase. The ligation products were transformed into *E. coli* competent cells. Typically, around 10–20 colonies would be obtained, which were generally enough for sub-sequential orientation and verification. The orientation and verification can be carried out by enzyme digestion, PCR and/or sequencing. For PCR and sequencing, both the GFP_Rev primer (Figure 1; Table S1) and promoter-specific primer could be employed.

### Genome-wide identification and expression patterns of pollen-specific genes

**Figure 3:**
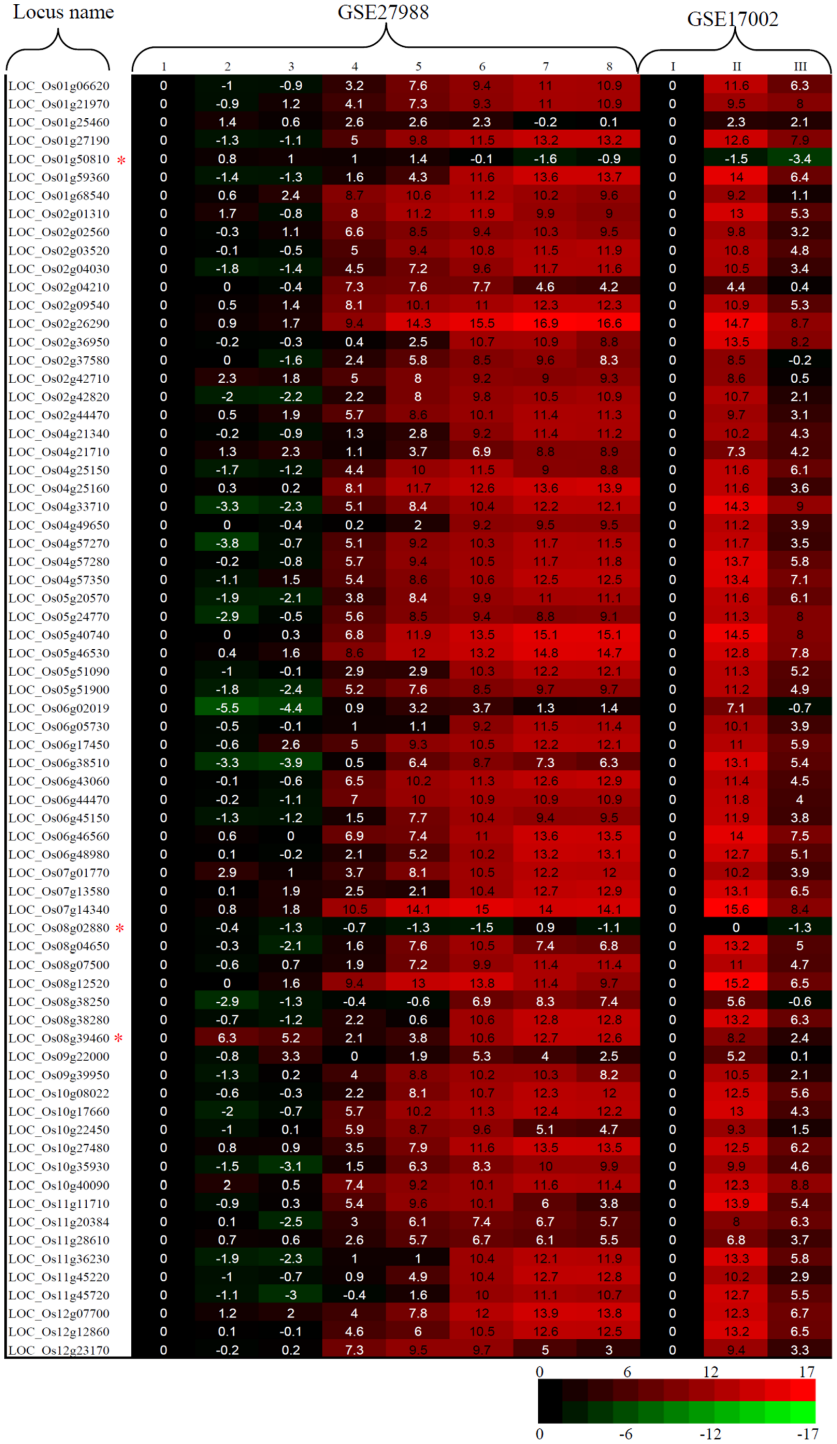
Expression patterns of pollen-specific genes by two sets of microarray data. The normalized microarray expression values in callus cells for the dataset GSE279881 and in seedlings for the dataset GSE17002 were set as controls, respectively. Their values were set as “1” and all other values were calculated by comparing with the control values. Then, all the values are log transformed (base 2 for simplicity) and the resulted log_2_ values were used for the heat mapping. Samples were labelled as below: 1, callus cells; 2, leaves; 3, roots; 4, uninucleate microspores; 5, bicellular pollens; 6, tricellular pollens; 7, mature pollen grains; 8, germinated pollen grains; I, seedlings; II, pollens at anthesis; III, sperms at anthesis. Red, black, and green colors indicated that transformed expression values were >0,  = 0, and <0, respectively, in the matrix. The red stars indicate the genes with inconsistent expression patterns when compared with the values from the MPSS dataset.

Based on the Massively Parallel Signature Sequencing (MPSS) rice database [39], a total of 1,013 pollen-specific genes were identified. These genes were then submitted to expression verification by microarray and RNA_Seq datasets. Among the 1,013 pollen-specific genes, no probe is available in the Affymetric array chips for 212 genes and we analyzed the expression patterns for the remaining 801 genes. By comparing two datasets, most of the genes showed pollen-specific expression. We were interested in highly expressed pollen-specific genes. A total of 70 genes with expression abundance more than 1,000 transcripts per million (TPM) in the MPSS database were selected for further analysis. Among these genes, both *LOC_Os01g50810* and *LOC_Os08g02880* were not pollen-specific and they showed similar or higher expression in seedling/roots than in pollens by microarray data analyses (Figure 3). For the gene *LOC_Os08g39460*, it was expressed in pollens with high expression level; however, it also showed high expression in callus and roots (Figure 3).

**Figure 4:**
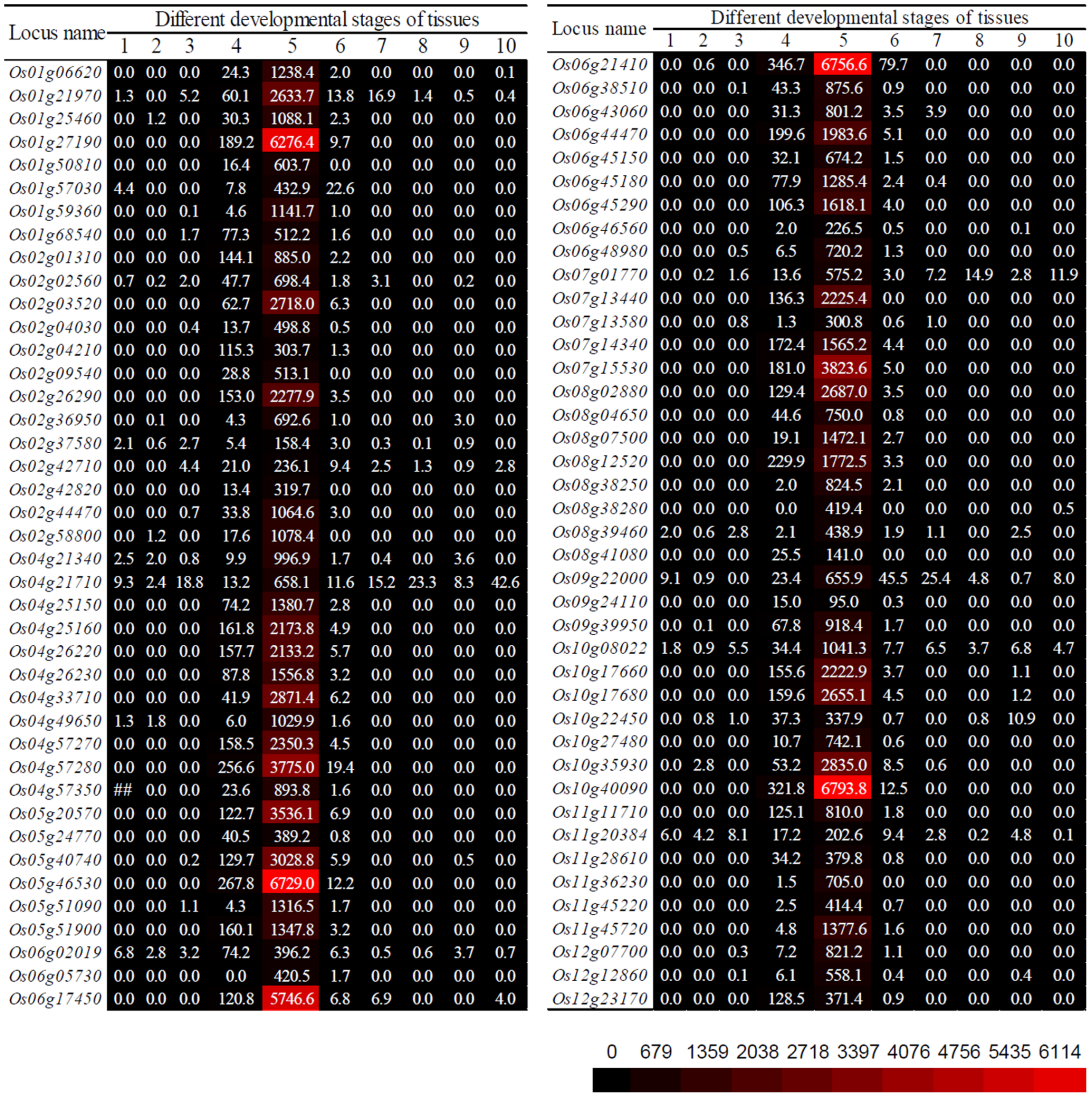
Expression heat map of pollen-specific genes by the RNA_Seq data. In the RNA_Seq dataset, a total of 10 different developmental stages of tissues were collected for the expression analysis. These tissues were labelled as below: 1, shoots; 2, leaves-20 days; 3, pre-emergence inflorescence; 4, post-emergence inflorescence; 5, anther; 6, pistil; 7, seed-5 days after pollination (DAP); 8, seed-10DAP; 9, embryo-25DAP; 10, endosperm-25 DAP. The normalized expression values were directly used to generate heat map. The values “0” indicated that no expression signal was detected for these genes in the corresponding tissues. The larger the expression values are, the stronger the genes show their expression. The prefix “LOC_” in locus names is omitted for convenience in this figure.

The RNA_Seq data (http://mpss.udel.edu/rice_RNAseq/) was then employed for further verification. In this database, expression data were available in 10 different tissues including shoots, 20-day old leaves, pre-emergence inflorescence, post-emergence inflorescence, anther, pistil, Seeds of 5-day after pollination (DAP) , 10 DAP seeds, 25 DAP embryos and 25 DAP endosperm. As 3 out of 70 genes showed non-pollen-specific expression by microarray data (Figure 3), these three genes were excluded for further verification by the RNA_Seq data. The verification analysis showed that, for all the 67 genes, the highest level of expression abundance was observed in the anthers (Figure 4). Further analysis showed that the expression signal of 97% genes in anthers is at least 5 times higher than the expression levels in the remaining tissues. The remaining 2 genes showed the expression level in anthers with 2.6 (*LOC_ Os02g04210*) and 2.9 (*LOC_Os12g23170*) times higher than in the other tissues, respectively (Figure 4). Thus, the RNA_Seq data further confirmed the pollen-specific expression of these 67 genes.

### Construction of binary vector carrying the promter::*GFP* cassette by using the novel T-vector

To further verify the pollen-specific expression of these candidate genes, we randomly selected one gene for promoter activity analysis. The selected gene is with the locus name *LOC_Os10g35930*. The 1,492 base pairs of DNA sequence upstream of start codon of the gene was amplified from the rice genome by PCR as shown in Figure 2. After verification by sequencing, the PCR fragment was directly ligated to the T-vector prepared by *Ahd*I digestion of pDsTGFP (Figure 2). After transformation, we have obtained a total of 9 colonies and five of them were randomly selected for plasmid DNA preparation and verification. Sequencing analysis showed that two colonies contained the insertion from the PCR fragment with correct orientation. One of the two colonies was selected for *Agrobacterium*-mediated genetic transformation to generate transgenic rice plants carrying the promoter::*GFP* cassette.

### The gene *LOC_Os10g35930* was expressed at the mature stage of panicle development

**Figure 5:**
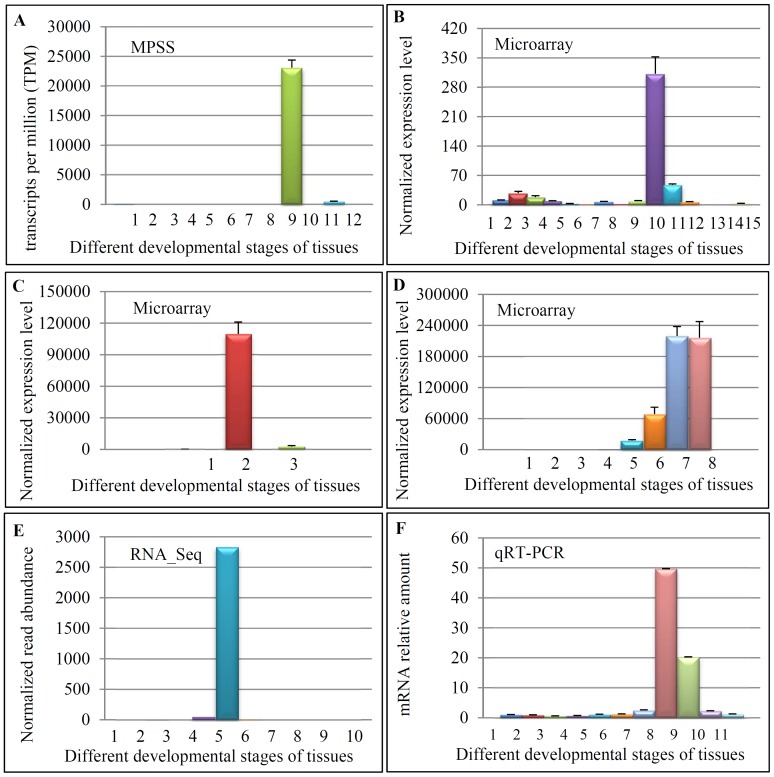
Detail analysis in the expression patterns of the gene *LOC_Os10g35930*. (A) The expression profile of the gene shown by the MPSS dataset. Samples were labeled as below: 1, 3-day old germinating seeds; 2, 10 days of germinating seedlings; 3, 14-day old young leaves; 4, 14day old young roots; 5, 60-day old mature leaves; 6, 60-day old mature roots; 7, 60-day old stems; 8, immature panicles; 9, mature pollens; 10, merismatic tissues; 11, ovary and mature stigmas; 12, rice developing seeds. (B) The gene showed mature inflorescence specific expression according to the microarray dataset with the GEO accession number GSE6893. Samples were labelled as below: 1, 7-day old roots; 2, mature leaves; 3, young leaves; 4, shoot apical meristem; 5, 0–3 cm inflorescence; 6, 3–5 cm inflorescence; 7, 5–10 inflorescence; 8, 10–15 cm inflorescence; 9, 15–22 cm inflorescence; 10, 22–30 cm inflorescence; 11, 0–2 day old seeds, 12, 3–4 day old seeds; 13, 5–10 day old seeds; 14, 11–20 day old seeds; 15, 21–29 day old seeds. (C) The gene showed the highest expression at mature pollens by the microarray dataset with the GEO accession number GSE17002. Samples were labelled as below: 1, seedling; 2, pollens at anthesis; 3, sperms at anthesis. (D) The gene showed the highest expression level at mature and germinated pollen grains. Samples were labelled as below: 1, callus cells; 2, leaves; 3, roots; 4, uninucleate microspores; 5, bicellular pollens; 6, tricellular pollens; 7, mature pollen grains; 8, germinated pollen grains. (E) The gene showed the anther-specific expression by the RNA_Seq data. The samples were labelled as below: 1, shoots; 2, 20 day old leaves; 3, pre-emergence inflorescence; 4, post-emergence inflorescence; 5, anthers; 6, pistil; 7, 5 day old seeds; 8, 10 day old seeds; 9, 25 day old seeds; 10, , 25 day old endosperms. (F) The gene showed the highest expression at the booting and flowering panicles by qRT-PCR. Samples were labelled as below: 1, two-week old leaves; 2, two-month old leaves; 3, two-week old roots; 4, two-month old roots; 5, 0–5 cm long panicles; 6, 5–10 cm long panicles; 7, more than 10 cm long panicles; 8, booting panicles; 9, flowering panicles; 10, milky seeds; and 11, mature seeds.

As the gene *LOC_Os10g35930* was selected as a candidate gene to further study its expression profiling by surveying its promoter activity, its expression patterns was also investigated in more details. The achieved data from the MPSS database showed that the gene *LOC_Os10g35930* was mainly expressed at the mature pollen grains (Figure 5A). We then analyzed the microarray data with GEO accession number GSE6893, where a total of 15 tissues were collected for expression analysis. The data showed that the gene *LOC_Os10g35930* has the highest expression signal at the last stage of inflorescence development, where most of pollens are in the mature stage (Figure 5B). The second microarray data consists of three tissues including seedling, pollens at anthesis and sperms at anthesis. The highest expression level was observed in the pollens at anthesis (Figure 5C). The third microarray data contained 8 samples and 5 of them were from pollens. The gene *LOC_Os10g35930* showed the highest expression level in both mature and germinated pollens (Figure 5D). Besides both the MPSS and microarray data, the RNA_Seq data (http://mpss.udel.edu/rice_RNAseq) was also employed for the expression verification (Figure 5E). Based on the data, the gene exhibited the strongest expression signal at the post-emergence inflorescence (Figure 5E). At this stage of inflorescence, most of the pollens were at the mature stage. Finally, the quantitative real-time reverse transcription polymerase chain reaction (qRT-PCR) was used to verify the expression of this gene. The total RNA samples were prepared from a total of 11 tissues and were then submitted to qRT-PCR analysis (Figure 5F). The analysis showed that the gene was mainly expressed at the opening panicles and flowering panicles, where majority of pollens were at the mature stage. Thus, our qRT-PCR data further confirmed the expression of this gene in the late stage of panicle development.

### The promoter activity in transgenic rice plants carrying the promoter::*GFP* cassette

**Figure 6:**
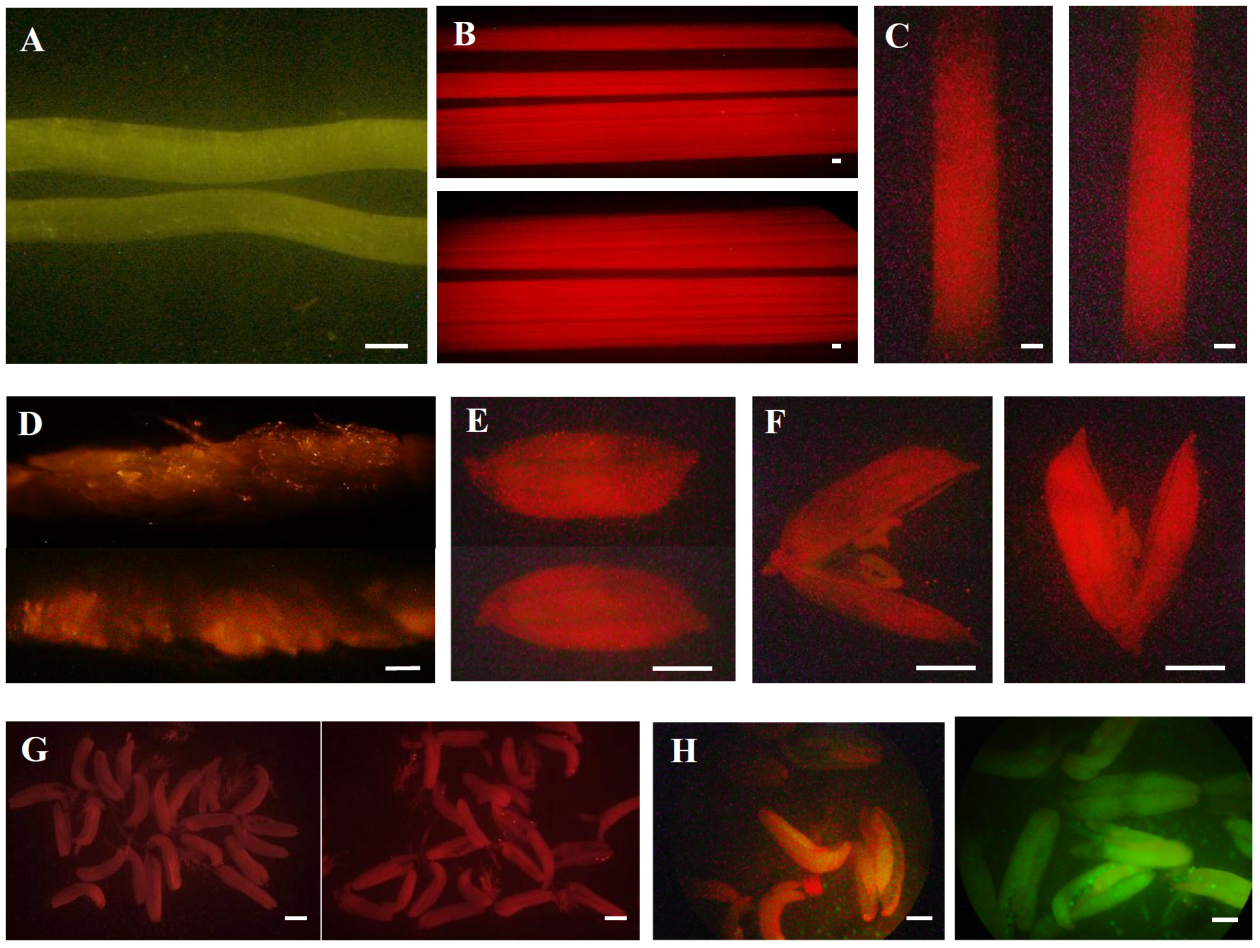
The GFP expression activities in various tissues shown by transgenic rice carrying the promoter::*GFP* construct. The 1,492 bp of promoter from the upstream of start codon of the gene *LOC_Os10g35930* was used to drive the expression of the reporter *GFP* gene. (A) No GFP signal in WT (top) and transgenic (bottom) roots. (B) No GFP expression in WT (top) and transgenic (bottom) leaves. (C) No GFP signal in WT (left) and transgenic (right) stems. (D) No GFP expression in WT (top) and transgenic (bottom) young panicles with less than 3 cm length. (E) No GFP expression in WT (top) and transgenic (bottom) young florets. (F) No GFP expression in WT (left) and transgenic (right) young anthers at the uninucleate microspore stage. (G) No GFP expression in WT (left) and transgenic (right) young anthers at the bicellular pollen stage. (H) No GFP expression in WT (left) and strong GFP signal in transgenic anthers (right) at the mature pollen stage. Bars = 1 mm.

The gene *LOC_Os10g35930* encodes a protein of 224 amino acids long with a molecular weight of 24.8 kDa. The domain searches in the Pfam database (http://pfam.xfam.org/) showed that the protein contained a LIM domain (PF00412). In order to investigate the expression activity of its promoter, the promoter::*GFP* transgenic plants were generated according to the description in the Methods section. Careful examination of GFP signal in the transgenic plants was carried out in the whole life cycle of rice development. No signal was detected in roots, leaves and stems (Figure 6 A–C) during the whole life cycle. Subsequently, the GFP signals in the florets at different developmental stages of panicles were examined. The results showed that no expression was observed at the early stages of panicles (Figure 6 D and E). Also, no signal was observed at the early stages of anther development (Figure 6 F and G). However, the strong GFP expression was observed at the mature stage of anther development (Figure 6H).

**Figure 7:**
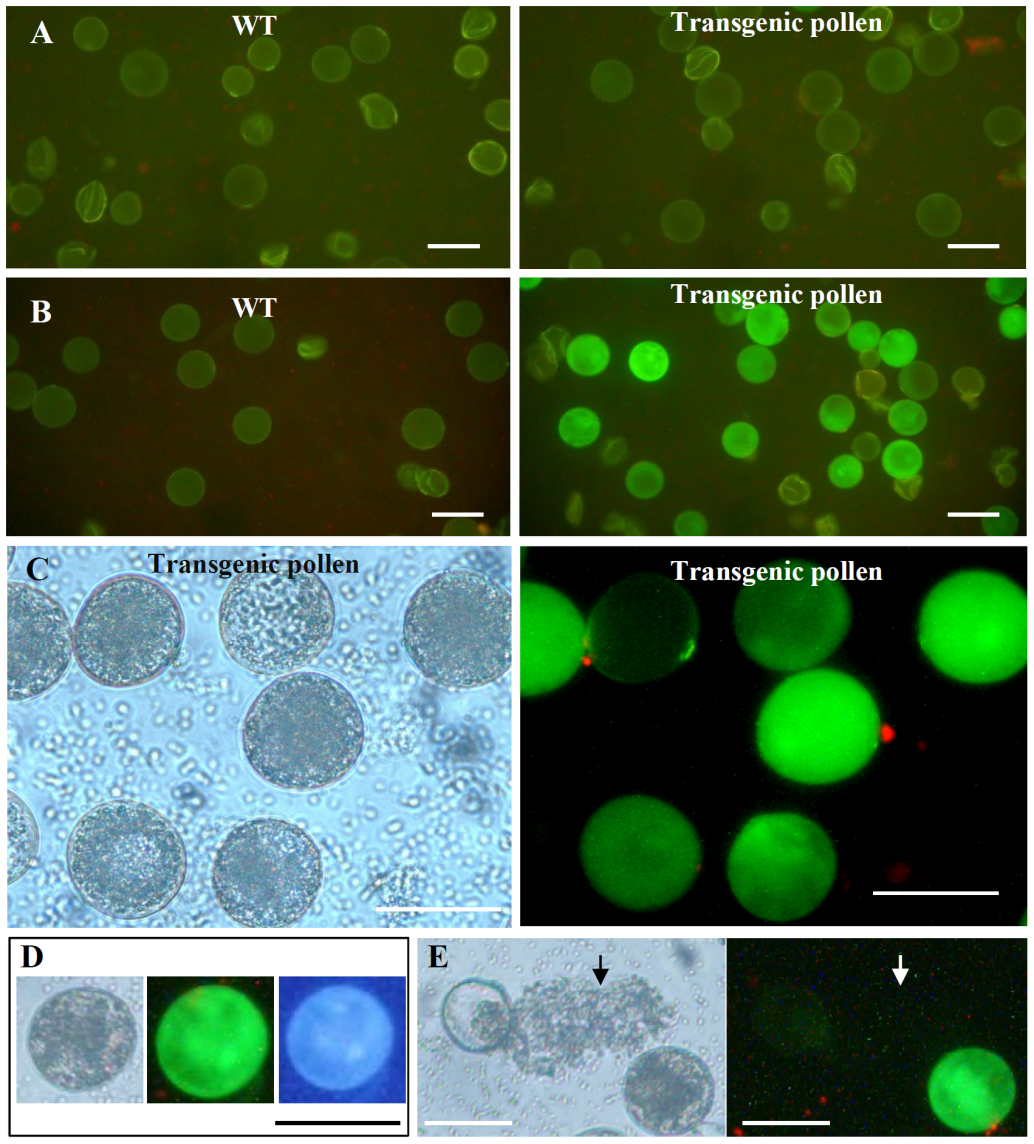
The GFP expression activites at various stages of pollens shown by transgenic rice carrying the promoter::*GFP* construct. (A) No GFP expression in WT (left) and transgenic (right) pollens at the uninucleate microspore stage. (B) No GFP expression in WT (left) and strong GFP expression in transgenic (right) tricellular pollens. (C) Enlarged images showing the transgenic mature pollens under visible light (left) and GFP signals under epifluorescence (right). (D) The left image shows mature pollen carrying the promoter::*GFP* cassette under visible light. The middle image shows GFP signal in the same pollen under epifluorescence and the right image shows DAPI staining at 3 nuclei in the pollen under UV light. (E) Germinated pollens under visible light (left) and their GFP expression level under epifluorescence (right). GFP signal was only observed in the mature pollen and no GFP signal was detected in a germinated pollen and its tube. Arrows indicate the germinated pollen tubes. Bars = 50 µm.

In order to investigate the expression activity of its promoter at the cellular level, various developmental stages of pollens were squeezed from anthers and were observed under microscope for GFP signal. No obvious GFP signal was observed at the unicellular stage of pollens when compared with the similar stage of pollens from the wild type plants (Figure 7A). However, at the mature stage of pollens, strong GFP signal was detected. The GFP signal was visualized in the whole area of pollens including membrane, cytoplasm and nucleus (Figure 7 B and C). The nucleic GFP signal was confirmed by DAPI (4′, 6-diamidino-2-phenylindole) staining at the tricellular stage of pollens (Figure 7D). In addition, the mature pollens harvested from the promoter-*GFP* transgenic plants were germinated *in vitro* and were then observed under microscope. The observation showed that the GFP signal was detected only in the mature pollens and no signal was visualized in the geminated pollens and their tubes (Figure 7E). Thus, our data suggested that the gene *LOC_Os10g35930* could be expressed only at the tricellular stage of pollens.

## Discussion

The novel T-vector is suitable for cloning PCR products amplified by Taq DNA polymerase which can catalyze the addition of an adenine residue to the 3′-end of its PCR fragment. If a PCR is carried out using other DNA polymerases, for example, Pfu DNA polymerase, the PCR products are required to be treated by Taq DNA polymerase to add A-overhang in both blunt ends for cloning. As only one overhang nucleotide at the ends of both PCR products and the T-vector, the DNA ligation and subsequent transformation should be carried out in more stringent conditions. Firstly, T-vector fragment should be properly purified. As the fragment is around 10 Kb in size, traditional methods used for gel purification of normal PCR products may not be suitable for the purification of the novel T-vector from agarose gel. Generally, acceptable cloning efficiency could be achieved when commercial columns suitable for large fragment DNA recovery were employed for T-vector purification from agarose gel. Secondly, low cloning efficiency may be observed when a fragment with larger than 2.5 Kb is used for cloning. Based on our experiment, relatively high efficiency could be achieved when PCR fragments ranging from 0.5–1.5 Kb were used for ligation. Since most of promoter sequences were less than 2 Kb, the novel T-vector should be applicable for cloning of most of promoters for the *GFP* reporter gene analysis. On the other hand, most of commercially available T-vectors are not binary vectors and they are not suitable for direct plant transformation. In addition, most of commercial T-vectors are only to provide multiple cloning sites flanking the insert for subcloning and no *GFP* gene is present in the T-vectors. Thus, our novel T-vector provides an alternative candidate for facilitating rapid construction of the promoter::*GFP* cassette for the reporter gene analysis.

Although many pollen-specific genes and their promoters have been identified and characterized as mentioned in the Introduction section, limited data are available on the genome-wide identification of pollen-specific genes. By using Affymetrix microarray rice chips, Wei *et al* (2010) revealed 25,062 pollen-preferential transcripts by investigating the expression profiles in three vegetative tissues and 5 pollen-related samples [40]. Genome-wide expression patterns among seedling, pollens and sperms at anthesis revealed 3,043 pollen-/sperm-specific genes [41]. In both cases, only one or three no-pollen tissues were selected for comparative expression analysis. Thus, some pollen-specific genes might be wrongly identified when these genes would be expressed in other vegetative tissues which were not be surveyed. Therefore, pollen-specific genes should be identified by using more vegetative tissues as references for more accurate evaluation. In this study, a total of 1,013 pollen-specific genes were identified by using the MPSS database. These genes were further verified in their expression microarray and RNA_Seq datasets and 67 highly expressed pollen-specific genes were selected for further analysis (Figures 3 and 4). As these pollen-specific genes were initially identified by using the MPSS database, where only the expression data from mature pollens are available, young pollen-specific genes were not included. Thus, more pollen-specific genes with high expression level should be identified if expression data from young pollens are available in the MPSS database.

A stage-specific/enriched gene is expressed only in a specific stage or shows significant higher abundance in the stage, for example, in the unicellular stage, of pollen development. The identification of the stage-specific/enriched genes should benefit to better understand the regulatory mechanism of pollen development. Wei *et al* (2010) identified 2,203 stage-enriched transcripts [40]. Another report showed that three transcriptomes of egg cells, sperm cells and pollen vegetative cells are highly divergent and about ¾ of those genes were differentially expressed in these cell types [42]. Russell *et al* (2012) also identified 1,668 sperm cell specific genes [41]. In this study, among 67 pollen-specific genes, most of them showed expression in the whole pollen development stages with the highest abundance in mature or germinated pollen grains and the expression level in sperms was obviously reduced (Figure 3). Our data suggested that most of mature pollen specific genes might play roles not only in mature pollens but also in their tube germination.

Previous studies revealed several pollen-specific promoter motifs [43]. We have selected 5 motifs for our further discussion. A total of 2,000 bp of promoter region in each promoter was selected to detect the presence of these motifs in the 67 putative pollen-specific promoters. Three of the five motifs, including “AGAAA” [44], “GTGA” [20] and “AAATGA” [24] could be detected in all the 67 promoter sequences. For the remaining two motifs “AGGTCA” [23] and “TGTGGTT” [45], 34% and 36% of the promoter sequences contained these two motifs, respectively. Most of these motifs were presented within 1.5 Kb upstream of the start codon of a gene. The data suggested that most of *cis*-elements to regulate pollen-specific expression should be located 1.5 Kb upstream region of a protein coding gene. Thus, in most of cases, 1.5 Kb of promoter region should be enough to drive pollen-specific expression.

Based on the expression patterns of the gene *LOC_Os10g35930* shown by microarray data, the inconsistence in expression patterns among different microarray datasets was observed. The gene was also expressed in both 0–2 day old seeds and germinated pollen grains with a detectable signal (Figure 5 B and D). However, only a very weak signal was observed in sperm cells (Figure 5C). Our data showed that no GFP signal was detected in these two tissues in the transgenic plants carrying the promoter::*GFP* construct (Figures 6 and 7), similar to the expression pattern as shown in Figure 5C. The weak inconsistence might be due to the methods used for sample collection in different experiments. On the other hand, promoter activity shown by the reporter *GFP* gene might be also affected by the promoter sequence length which was used to construct the promoter::*GFP* cassette.

## Materials and Methods

### Plant materials and growth conditions

The japonica rice variety Nipponbare plants (*Oryza sativa* L.) were used for all DNA and RNA preparation as well as *Agrobacterium*-mediated genetic transformation in this study. The rice seeds were germinated in water at 37°C for three days and were then planted in pots. All the plants were grown in greenhouse under natural light and temperature conditions.

### Preparation of DNA and total RNA samples

Leaves from two-week old plants were collected and were then frozen in liquid nitrogen for DNA isolation as described by Dellaporta *et al* (1983) [46]. For total RNA isolation, a total of 11 different stages of rice tissues were collected and these tissues were listed as below: (1) two-week old leaves, (2) two-month old leaves, (3) two-week old roots, (4) two-month old roots, (5) 0–5 cm long panicles, (6) 5–10 cm long panicles, (7) more than 10 cm long panicles, (8) booting panicles, (9) flowering panicles, (10) milky seeds and (11) mature seeds. Total RNA samples were isolated using a QIAGEN RNeasy Mini kit. The total RNA quality was analysed by Nanodrop reading and only the total RNA samples with A260/A280> = 1.8 were used for further experiments.

### PCR amplification of bridge fragments from the rice genome for the construction of the T-vector pDsTGFP

In order to introduce two *Ahd*I restriction sites into the pCAMBIA 1300 vector, two DNA fragments were amplified from the rice genome using two primer sets as described in Table S1. One of the fragments (425 bp) matches the region from 77322^nd^–77746^th^ of the rice BAC clone OSJNBb0049H14 and another fragment (900 bp) is from the region 76044^th^–76943^rd^ of the rice PAC clone P0624H09. PCR amplification was carried out in 25 µl of reaction mixtures with 50 ng of genomic DNA, 200 µM of each of dNTPs, 0.5 µM each of primers, 2.5 mM MgCl_2_, 1 unit of DNA Taq polymerase from QIAGEN, and buffer provided by the supplier. The temperature profile started at 94°C for 5 min and followed by 30 cycles at 94°C for 40 s, 55°C–65°C for 40 s (depending on the Tm value of primers) and 72°C for 1 min. The reaction was terminated at 72° for 10 min. After amplification, the two fragments were purified from agarose gel and were then cloned into pGEM-T Easy Vector from Promega. These two fragments were then subcloned into the *GFP* containing binary vector pCAMBIA 1300 by multiple steps of enzyme digestion and ligation.

### Expression datasets used in this study for expression verification of pollen-specific genes

The MPSS dataset was downloaded from the website https://mpss.udel.edu/rice/mpss_index.php [39]. Three microarray datasets were downloaded from the GEO datasets [36] (http://www.ncbi.nlm.nih.gov/geo/) with accession numbers GSE6893, GSE17002 and GSE27988. The RNA_Seq dataset was downloaded from the MSU rice genome annotation database [47] (http://rice.plantbiology.msu.edu/index.shtml).

### Expression analysis by qRT-PCR

The primer sequences used for qRT-PCR were selected by the Applied Biosystems Primer Express software and were listed in Table S1. An *eEF-1a* gene from the rice genome was used as an internal control to normalize the amplification data. Their primer sequences were also listed in Table S1. A total of 11 RNA samples from different tissues were submitted to the first-strand cDNA synthesis using an Invitrogen kit. The cDNA first strand was used as templates for qRT-PCR analyses. The reactions were carried out using the AB power SYBR Green PCR Master mix kit (Applied Biosystems, P/N 4367659) according to the manufacturer's protocol. The threshold cycle (C_T_) value was automatically calculated based on the fluorescence of SYBR Green I dye in every cycle, which was monitored by the ABI 7900 system software. The mRNA relative amount was calculated by ΔΔC_T_ according to our previous description [48]. The value was used to evaluate the expression profiling of a gene.

### The construction of the promoter::*GFP* cassette and *Agrobacterium*-mediated transformation

The gene *LOC_Os10g35930* shows pollen-specific expression and its promoter was selected as an example to construct the promoter::*GFP* cassette using the novel T-vector pDsTGFP. A 1,492 bp of promoter fragment was amplified by PCR using the promoter-specific primer set as listed in Table S1. After purification from agarose gel and sequencing verification, the promoter fragment was directly ligated to the T-overhang pDsTGFP. The ligation was then used for transformation into the competent *E. coli* DH5α cells by the heat-shock method.

Plasmid DNA samples were prepared using QIAGEN Plasmid Mini Kit. After verification and orientation by PCR and sequencing, the plasmid DNA was then introduced into *Agrobacterium* AGL1 by electroporation using GIBCOBRL Cell-Porator. Embryonic calli were induced from mature rice embryos and were used for *Agrobacterium*-mediated genetic transformation according to the description by Hiei *et al* (1994) [49].

### Fluorescence microscopy for GFP signal detection

A Nikon microscope with epifluorescence was used to detect the promoter-driven GFP expression using a B1E filter (excitation 470 to 490 nm, dichronic mirror 515 nm and barrier filter 520 to 560 nm). Photos were taken with the attached digital imaging system.

### Sequencing of DNA fragments

DNA sequencing reactions were performed using the ABI PRISM Big Dye Terminator Cycle Sequencing kit according to the instruction from the supplier. The PTC200 (MJ Research, Inc.) thermocycler was used for temperature profiling according to the ABI cycle sequencing protocol: 25 cycles at 96°C for 30 s, 50°C for 15 s and 60°C for 4 min. The sequencing samples were purified using the Agencourt CleanSEQ sequencing reaction clean-up system (Beckman Coulter) and was then analyzed using an ABI 377 automatic sequencer.

## Supporting Information

Table S1 **Primer sequences used for PCR, sequencing and qRT-PCR.**
(XLSX)
